# Quantitative and Chemical Fingerprint Analysis for the Quality Evaluation of *Isatis indigotica* based on Ultra-Performance Liquid Chromatography with Photodiode Array Detector Combined with Chemometric Methods

**DOI:** 10.3390/ijms13079035

**Published:** 2012-07-20

**Authors:** Yan-Hong Shi, Zhi-Yong Xie, Rui Wang, Shan-Jun Huang, Yi-Ming Li, Zheng-Tao Wang

**Affiliations:** 1 School of Pharmacy, Shanghai University of Traditional Chinese Medicine, Shanghai 201203, China; E-Mails: yhs_lucky@163.com (Y.-H.S.); xzy0372@yahoo.com.cn (Z.-Y.X.); sese1986@gmail.com (S.-J.H.); ymlius@163.com (Y.-M.L.); 2 Institute of Chinese Materia Medica, Shanghai University of Traditional Chinese Medicine, Shanghai 201203, China; E-Mail: wangzht@hotmail.com

**Keywords:** *Radix Isatidis*, UPLC-PDA, quality control, quantitative, fingerprint

## Abstract

A simple and reliable method of ultra-performance liquid chromatography with photodiode array detector (UPLC-PDA) was developed to control the quality of *Radix Isatidis* (dried root of *Isatis indigotica*) for chemical fingerprint analysis and quantitative analysis of eight bioactive constituents, including *R*,*S*-goitrin, progoitrin, epiprogoitrin, gluconapin, adenosine, uridine, guanosine, and hypoxanthine. In quantitative analysis, the eight components showed good regression (*R* > 0.9997) within test ranges, and the recovery method ranged from 99.5% to 103.0%. The UPLC fingerprints of the *Radix Isatidis* samples were compared by performing chemometric procedures, including similarity analysis, hierarchical clustering analysis, and principal component analysis. The chemometric procedures classified *Radix Isatidis* and its finished products such that all samples could be successfully grouped according to crude herbs, prepared slices, and adulterant *Baphicacanthis cusiae* Rhizoma et Radix. The combination of quantitative and chromatographic fingerprint analysis can be used for the quality assessment of *Radix Isatidis* and its finished products.

## 1. Introduction

*Radix Isatidis* is the dried root of *Isatis indigotica* Fort. (Fam. Cruciferae), which is known as *Banlangen* (BLG) or *Bei-Banlangen. Baphicacanthis cusiae* Rhizoma et Radix is the dried rhizome and root of *Baphicacanthus cusia* (Nees) Bremek. (Fam. Acanthaceae), which is known as *Nan-Banlangen* (NBLG). In several southern regions in China, NBLG has been improperly used as *Radix Isatidis* even though both have been officially listed in Chinese Pharmacopoeia (State Pharmacopoeia 2000) as two different crude herbs [1–4].

*Radix Isatidis* and its finished products have important functions in preventing and treating influenza, tonsillitis, and malignant infectious diseases [5–7], especially severe acute respiratory syndrome (SARS) and H1N1-influenza [8–11] because of its anti-viral, anti-bacterial, anti-inflammatory, anti-tumor, and immune regulatory functions. Moreover, numerous gratifying successes of the *Radix Isatidis* anti-viral effect have been reported [12–15]. *Radix Isatidis* has become an important component in various traditional Chinese medicine preparations (TCMPs), of which Banlangen Granules (BLGG) is widely used for toxic-heat removal and as an anti-viral drug in clinical practice.

The chemical constituents in *Radix Isatidis* are as follows: nucleosides, glucosinolates, amino acids, polysaccharides, alkaloids, organic acid, trace elements, and so on [16–18]. Compared with other *Radix Isatidis* extraction methods, the water extraction method has the most significant anti-viral, anti-bacterial, and anti-endotoxic effects, and the chemical constituents of glucosinolates (*R*,*S*-gotrin, progoitrin, epiproguotrin, and gluconapin) and nucleosides (hypoxanthine, adenosine, uridine, and guanosine) are the major bioactive components [19–21]. Therefore, using the non-polar components of Indigotin, Indirubin, and the nonspecific amino acid constituent as quality control markers of *Radix Isatidis* is unsuitable [22–27].

Glucosinolates are one of the characteristic components of Cruciferae. They degrade under endogenous myrosinase effects to work on multi-bioactivities including anti-virus and anti-bacteria [28,29]. *R*,*S*-goitrin reflects bioactivities that are relevant to the *Radix Isatidis* effects with high specificity. In our previous studies, we established thin layer chromatography (TLC) identification and high-performance liquid chromatography (HPLC) assay methods for the quality control of *Radix Isatidis* using *R*,*S*-goitrin as the marker [30], and these methods have been adopted by the ChP 2010 Edition [1]. Progoitrin, epiproguotrin, and *R,S*-gotrin all exist in *Radix Isatidis*. Under the effects of myrosinase, some parts or all of progoitrin and epiproguotrin transfer to *R*,*S*-goitrin via degradation [31].

In previous studies, based on the issues related to *Radix Isatidis* such as the improper use of NBLG, glucosinolate degradation, lack of specificity using amino acid as the BLGG quality control marker, establishing proper and scientific-based methods for quality control of *Radix Isatidis* and its finished products is necessary. Current studies about quantitative and chemical fingerprinting of *Radix Isatidis* depend on non-polar extracts or spots of chemical compositions [32–35]. Chemical fingerprint and quantitative analysis has become one of the most frequently applied approaches in the quality control of traditional Chinese medicine (TCM) and its finished products [36–41]. Studies about combining chromatographic fingerprint and multi-ingredient quantification by ultra-performance liquid chromatography with photodiode array detector (UPLC-PDA) for the quality control of *Radix Isatidis* and its preparations have not been reported.

This study aims to reveal the correlation and consistency of quality control in crude herbs, prepared slices, and *Radix Isatidis* preparations. A simple, accurate, and practical UPLC-PDA method was developed for the simultaneous determination of eight bioactive components in *Radix Isatidis* and its preparations. The chemical fingerprints of *Radix Isatidis* from various sources were established and investigated by similarity analysis (SA), hierarchical clustering analysis (HCA), and principal component analysis (PCA). The combination of chromatographic fingerprint analysis and the simultaneous determination of the eight bioactive components offer a more comprehensive strategy for the quality evaluation of *Radix Isatidis* and its finished products.

## 2. Results and Discussion

### 2.1. Optimization of UPLC Conditions

Different UPLC parameters were examined and compared, including various columns, mobile phases, detection wavelengths, and gradient elution conditions to obtain as much chemical information as possible and to determine the best separation mechanism in chromatograms.

Four kinds of reversed-phase columns, namely, Waters ACQUITY UPLC BEH C_18_ (1.7 μm, 2.1 mm × 50 mm/100 mm) and Thermo Syncronis C_18_/Waters ACQUITY UPLC HSS T_3_ (1.7 μm, 2.1 mm × 100 mm) were investigated and compared. The Waters ACQUITY UPLC BEH C_18_ (1.7 μm, 2.1 mm × 100 mm) column had good peak separation and sharp peaks.

The effect of mobile phase composition (methanol-water and acetonitrile-water with different modifiers including acetic acid, formic acid, phosphoric acid, and triethylamine) on chromatographic separation was investigated. Adding 0.1% triethylamine in the mobile phase and adjusting the pH to 4.0 with formic acid provided a better resolution and separation of the eight bioactivity components, and resulted in high precision sensitivity and selectivity.

**Figure 1:**
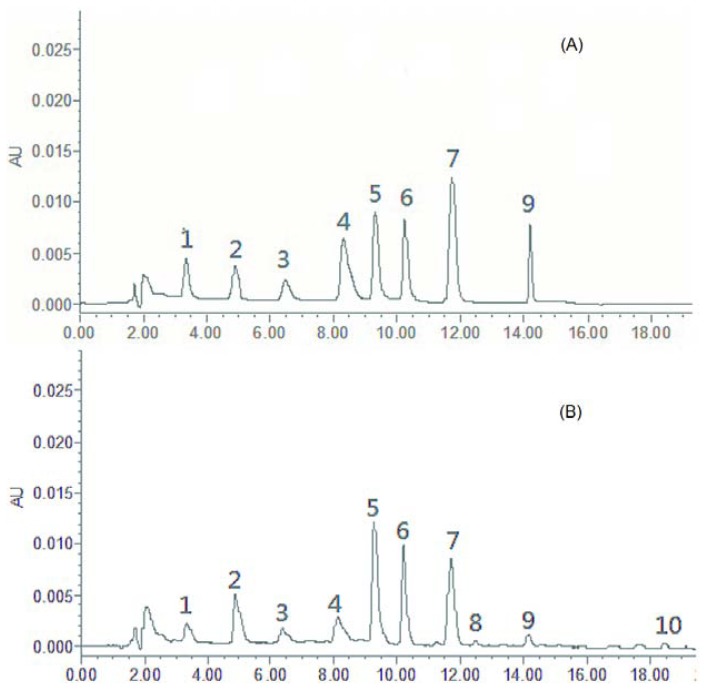

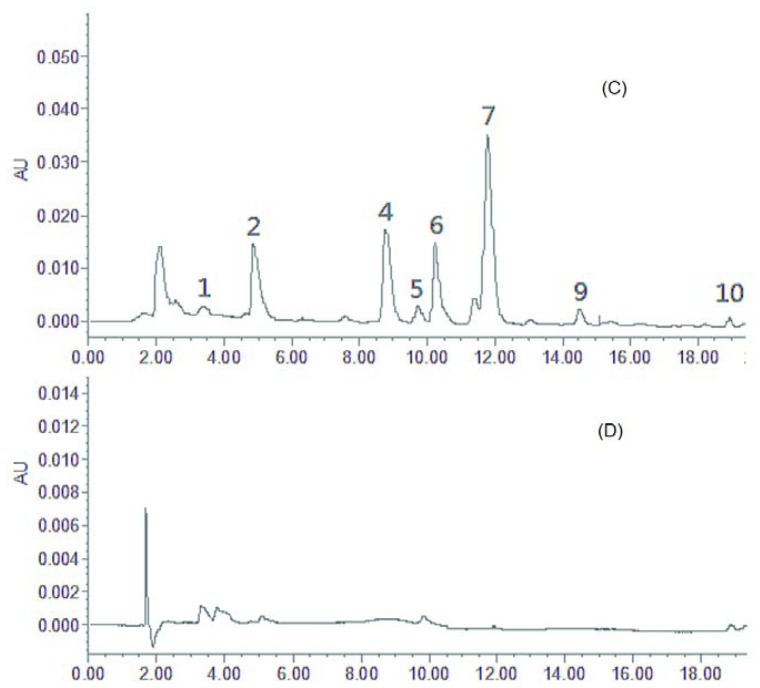
Typical chromatograms for determining the eight bioactive compounds in different samples. (**A**) Mixed standards; (**B**) *Radix Isatidis* (No. 20101121); (**C**) Banlangen Granules (BLGG) (No. 090921); (**D**) Nan-Banlangen (NBLG) (No. 060308-1). Peaks 1 = hypoxanthine, 2 = uridine, 3 = progoitrin, 4 = epiprogoitrin, 5 = adenosine, 6 = guanosine, 7 = *R,S*-goitrin, and 9 = luconapin.

Based on the maximum absorption and full-scan experiment of the marker components in the UV spectra of the three-dimensional chromatograms obtained by PDA detection, the detection wavelengths at 210, 230, 254, 280 nm were selected to compare the peak number and peak resolution of all marker compounds. Finally, the wavelength was set at 254 nm (Figure 1).

### 2.2. Optimization of Extraction Methods

Satisfactory extraction efficiency was obtained by comparing water-refluxing, ultrasonic, and soxhlet extraction methods. Refluxing extraction was simpler and more effective for nucleosides and glucosinolates among the other methods. Therefore, this method was used in further experiments. In this study, different concentrations (0%, 20%, 50%, 75%, 90%, and 100%) of ethanol solutions, sample-solvent ratios (1:10, 1:20, 1:50, and 1:100, *w*/*v*), and extraction times (20, 30, 45, and 60 min) were used for the BLG extraction procedure (Batch No. 20101121). As a result, the best extraction condition was established as follows: the samples were extracted by refluxing extraction using 20 mL of water as the extraction solvent, and the duration was 30 min.

### 2.3. Method Validation of Quantitative Analysis

The method was validated in terms of linearity, limit of detection (LOD), limit of quantification (LOQ), precision, reproducibility, stability, and recovery test.

#### 2.3.1. Calibration Curves, LOD, and LOQ

Methanol stock solutions containing eight analytes were diluted to appropriate concentrations for calibration curve construction. The analyte solutions at six different concentrations were injected in triplicate, and the calibration curves were established by plotting the peak area (*Y*) versus the concentration (*x*) of each component. LOD and LOQ, which were expressed by 3-and 10-fold of the signal-to-noise ratio (*S*/*N*), were also determined. The detailed information regarding the calibration curves, linear ranges, LODs, and LOQs of the eight bioactive compounds are listed in Table 1.

#### 2.3.2. Precision, Reproducibility, Stability, and Recovery

As shown in Table 2, the precision based on the peak area measurements of the eight bioactive components were higher than 0.48% (RSD, *n* = 6, S-01). The reproducibility (RSD, *n* = 6, S-01) of the proposed method based on six replicate injections was in the range of 0.10% to 1.24%. The stability (RSD, *n* = 6, S-01) of the measurements over 3 days for the eight compounds was 0.22% to 1.44%. The recovery test was performed by the standard addition method. Low, medium, and large high amounts of the standards were added to the known sample (S-01). Extraction and analysis were performed as described in Section 2.4. The mean recovery was calculated according to the following formula: recovery (%) = (amount found–original amount)/amount spiked × 100%, and RSD (%) = (SD/mean) × 100%. The mean recovery of the eight bioactive compounds was 99.5%–103.0%, and the RSD value was 0.73%–1.81%.

### 2.4. Sample Analysis

The newly established analytical method was subsequently applied to determine simultaneously the eight bioactive components in 21 commercial samples of *Radix Isatidis* and its finished products from different provinces or manufacturers in China. All samples were analyzed using the optimized extraction method in optimized UPLC conditions. Each sample was analyzed in triplicate to determine the mean content (mg·g^−1^), and the results are tabulated in Table 3.

**Figure 2:**
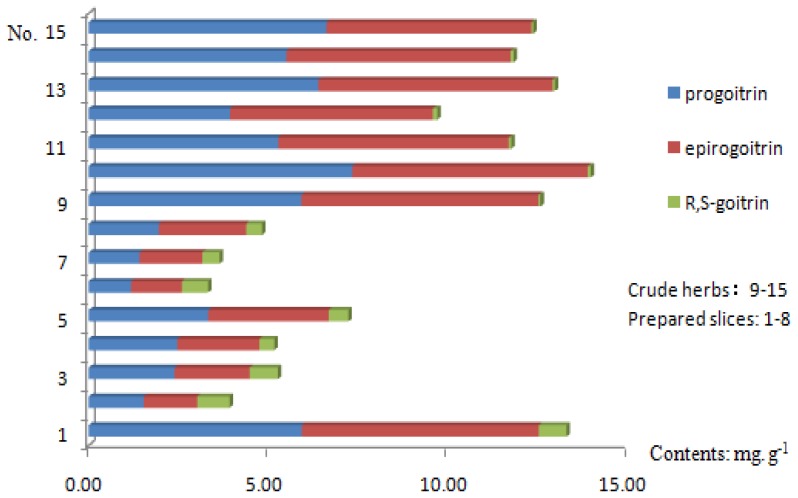
Bar graph of the contents of progoitrin, epiprogoitrin, and *R*,*S*-gotrin in 15 sample batches.

Glucosinolates are the characteristic constituents in the Cruciferae plants [28,29]. The results from the quantitative analysis (Table 3) showed that the crude herbs generally contained the eight selected constituents and the glucosinolate content ranged from 6.48 mg g^−1^ to 73.63 mg g^−1^. Table 3 and Figure 2 show that the average contents of epiprogoitrin, progoitrin, and *R,S*-goitrin in *Radix Isatidis* crude herbs are 5.86, 6.25, and 0.07 mg g^−1^. However, the average epiprogoitrin and progoitrin contents in prepared *Radix Isatidis* slices significantly decreased (2.53 and 2.68 mg g^−1^, respectively), whereas the *R*,*S*-goitrin content clearly increased (0.64 mg g^−1^). The traditional processing methods improve the glucosinolate biotransformation and increase the degradation product (*R*,*S*-goitrin) content [31].

The total content of eight compounds in different samples, especially those from different sources and harvesting times, were significantly different. Moreover, epiprogoitrin and progoitrin could not be distinctly detected (below LOD) in BLGG that was prepared and precipitated with ethanol by the water extracts of *Radix Isatidis* [1].

Overall, the internal qualities of 21 batches of *Radix Isatidis* samples from different sources with different geographical sources were varied, and the qualities needed evaluation by chemical fingerprinting.

### 2.5. UPLC Fingerprint of *Radix Isatidis*

**Figure 3:**
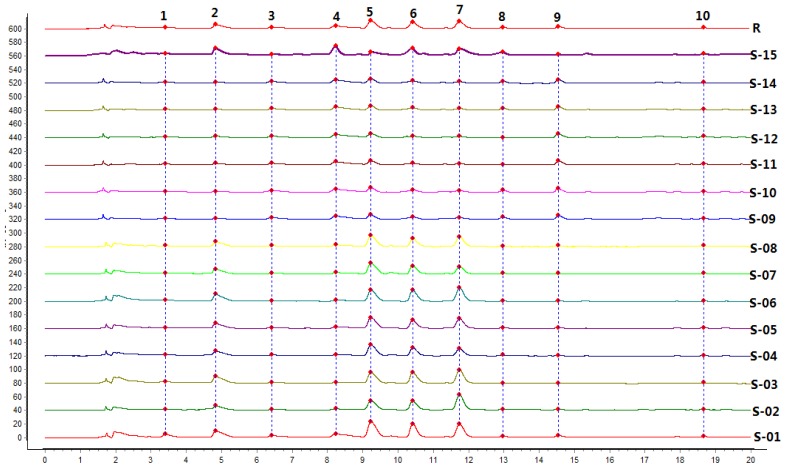
Ultra-performance liquid chromatography (UPLC) fingerprint chromatograms of 15 batches of *Radix Isatidis* (R: digital standard fingerprint).

Altogether, 15 batches of samples were analyzed, and all chromatograms were introduced into the Computer-Aided Similarity Evaluation System for Chromatographic Fingerprint of TCM (China Committee of Pharmacopeia, 2004). Peaks existing in all sample chromatograms were assigned as the “common peak,” and 10 common peaks were observed between 3 and 20 min in all 15 batches (Figure 1). Eight common peaks (peak 1, 2, 3, 4, 5, 6, 7, and 9) were identified as hypoxanthine, uridine, progoitrin, epiprogoitrin, adenosine, guanosine, *R,S*-goitrin, and gluconapin with reference substances (Figure 3). Peak 7 (*R*,*S*-goitrin, RT = 11 min), which was one of the most important active constituents of *Radix Isatidis* (China Pharmacopoeia Committee 2010), was chosen as the internal reference peak to calculate the relative retention time (RRT) and relative peak area (RPA) of the other peaks.

Figure 1 shows that the investigated nucleosides, glucosinolates, and other compounds in *Radix Isatidis* were separated and determined using the developed UPLC-PDA method. The differences of the water-soluble bioactive components from BLG and NBLG were recognized, and the components could be rapidly and efficiently differentiated by the chromatographic method (Figure 1B,D). Moreover, the bioactive constituents of glucosinolates and nucleosides were simultaneously shown by the same chromatographic method, and the high correlation between BLG and BLGG (Figure 1B,C) provided a basis to investigate the relationship between *Radix Isatidis* and its preparations.

#### 2.5.1. Similarity Analysis (SA)

The State Food and Drug Administration (SFDA) suggested that all herbal chromatograms should be evaluated in terms of similarity by calculating the correlation coefficient and/or angle cosine value of the original data [36–38]. Therefore, SA was conducted based on the standard fingerprints, and the results are shown in Table 4. The same samples showed similarities (crude herbs: 0.752 to 0.820; prepared slices: 0.933 to 0.991). The same samples had similar constituents with the slight difference resulting from the environmental conditions and the planting techniques. However, the crude herbs and the prepared slices were significantly different because of the production process and the contents of main bioactive constituents. The biotransformation, the glucosinolate contents, and the degradation products have the most important influence on the similarities.

#### 2.5.2. Hierarchical Cluster Analysis (HCA)

**Figure 4:**
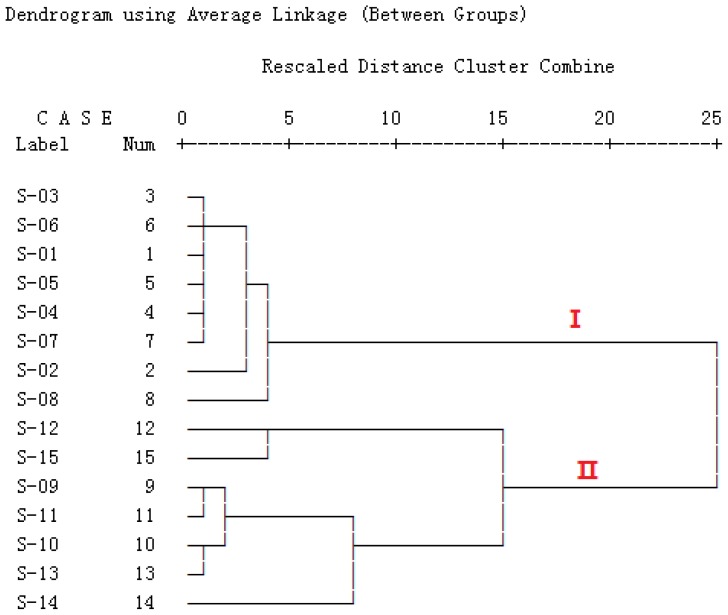
Dendrogram of hierarchical clustering analysis (HCA) for the 15 tested samples. The hierarchical clustering was done by the SPSS software (version 15.0; IBM: Chicago, IL, USA, 2006). Ward’s method was performed, and Squared Euclidean distance was selected as a measurement.

The HCA results clearly showed that the samples were appropriately divided into two main clusters related to the *Radix Isatidis* type (Figure 4). Cluster-I was S01 to S08, which were the prepared slices of *Radix Isatidis*, whereas Cluster-II was S09 to S15, which were the crude herb samples. The HCA result was fully consistent with the actual situation of samples.

#### 2.5.3. Principal Component Analysis (PCA)

**Figure 5:**
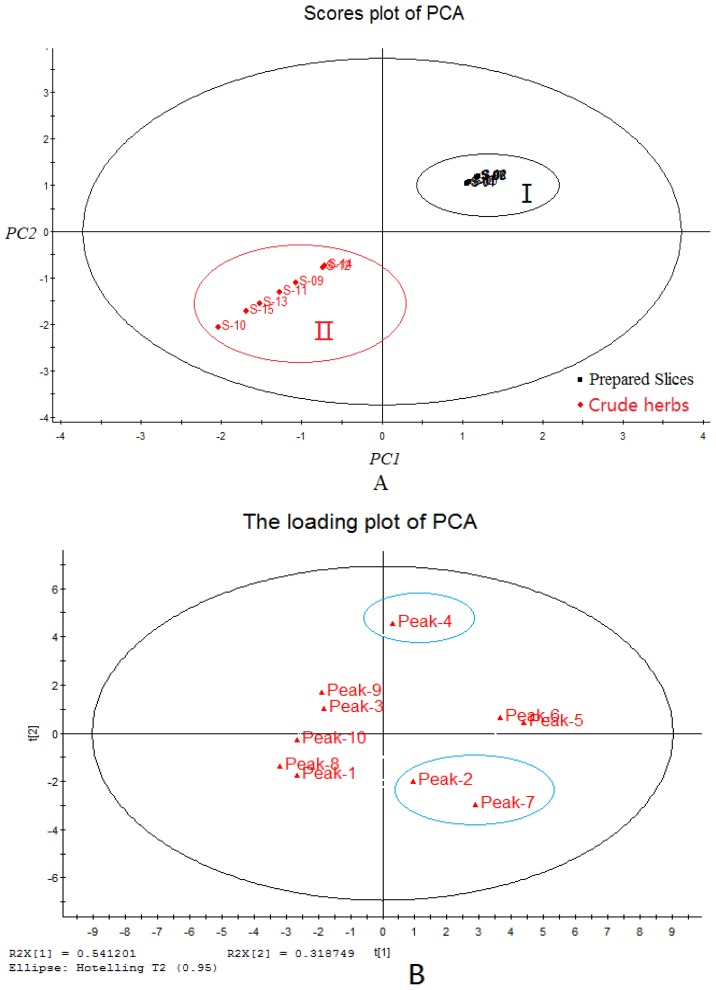
Score plot of principal component analysis (PCA) (**A**) and PCA loading plot (**B**) of *Radix Isatidis*.

PCA, which is an unsupervised multivariate data analysis approach, is appropriate when a function of many attributes is believed to be involved in different samples [40,41]. PCA was employed to analyze the relationship of the 15 *Radix Isatidis* samples (seven crude herbs and eight prepared slices) from different sources, and the score plot derived from PCA is shown in Figure 5A.

On the basis of eigenvalues > 1, the first two principal components, PC1 and PC2, are often used to provide a convenient visual aid for identifying inhomogeneity in the data sets. The samples were clustered into two main groups. The PCA loading plot (Figure 5B) indicated that peak 4 (progoitrin) and peak 7 (*R*,*S*-goitrin) showed the greatest influence on the scores. Peak 2 (uridine) and other compounds also affected the scores. Progoitrin content in the crude herbs was obviously higher than that in the prepared slices, whereas *R*,*S*-goitrin content had a reverse trend. The difference between crude herbs and prepared *Radix Isatidis* slices could be generally tracked to the transformation of glucosinolates to their degradation products.

## 3. Experimental Section

### 3.1. Materials, Reagents, and Chemicals

Eight BLG (prepared slices) batches, seven BLG (crude herbs) batches, six BLGG batches, and four NBLG batches were collected from different provinces or manufacturers in China, and were numbered from S-01 to S-25 (Table 4). The BLG and NBLG samples were identified by Dr. Li-Hong Wu, and the voucher specimens were deposited in the Herbarium of Shanghai University of Traditional Chinese Medicine (Shanghai, China).

**Figure 6:**
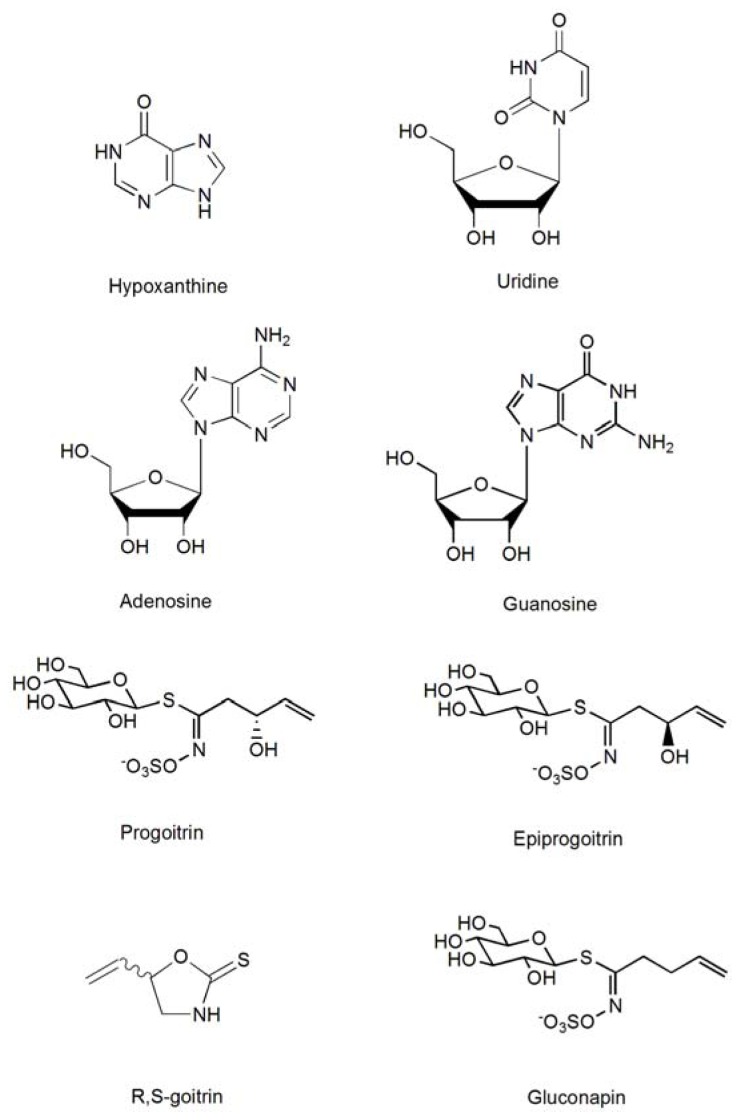
The structures of eight bioactive components in *Radix Isatidis.*

*R*,*S*-goitrin was obtained from Shanghai Research and Development Center for Standardization of Traditional Chinese Medicine (Shanghai, China). Hypoxanthine, uridine, guanosine, and adenosine were purchased from the National Institute for Food and Drug control (Beijing, China). Progoitrin, epiprogoitrin, and gluconapin were isolated from the root of *Isatis indigotica* Fort. and their structures were elucidated by mass spectrometry, ^1^H nuclear magnetic resonance spectrometry, and ^13^C nuclear magnetic resonance spectroscopy, and were confirmed by comparing the data with those of previous studies. The compound purity was over 98% based on the HPLC area normalization method. The standard structures are illustrated in Figure 6.

HPLC-grade acetonitrile, triethylamine, and formic acid (Tedia, Fairfield, OH, USA) were used for mobile phase preparation. Purified water was prepared with a Mili-Q water-purification system (Millipore, Bedford, MA, USA). Other solvents used for analyses were of analytical grade.

### 3.2. Instrumentation and Chromatographic Condition

UPLC analysis was performed on a Waters ACQUITY UPLC H-Class system (Waters, Milford, MA, USA) equipped with a binary solvent delivery pump, an auto sampler, and a photodiode array detector (PDA) and was controlled by the Empower-II software. UPLC fingerprinting analysis was carried out at 25 °C on a Waters ACQUITY UPLC BEH C_18_ (1.7 μm, 2.1 mm × 100 mm). A binary gradient elution system composed of acetonitrile (phase A) and 0.1% triethylamine in water (phase B, the pH was adjusted to 4.0 with formic acid) was applied to the fingerprint analysis with the gradient elution as follows: 0 min to 5 min, 0% A; 5 min to 7 min, 0% to 3% A; 7 min to 10 min, 3% A; 10 min to 20 min, 3% to 10% A. The wavelength was set at 254 nm, the mobile flow rate at 0.3 mL min^−1^, and the on-line UV spectra was recorded in the range of 190 nm to 500 nm.

### 3.3. Preparation of Standard Solution

*R*,*S*-goitrin, progoitrin, epiprogoitrin, gluconapin, adenosine, uridine, guanosine, and hypoxanthine CRS were accurately weighed, and dissolved in methanol to produce a solution containing 1.0 mg mL^−1^, which was used as the reference solution.

### 3.4. Preparation of Sample Solution

The crude herbs and the prepared BLG and NBLG slices were oven-dried at 50 °C until the weight remained constant. Each sample (1.0 g powder) was extracted with 20 mL of water, which was subjected to reflux for 30 min. The final solution was filtered through a 0.22 μm membrane prior to use. An aliquot of 2.0 μL of each sample solution was injected into the UPLC system for analysis.

### 3.5. Method Validation

The method was validated for linearity, LOD, LOQ, precision, reproducibility, stability, and accuracy following the International Conference on Harmonization (ICH) guideline and several reports on determination analysis [36,38].

### 3.6. Data Analysis

Data analysis was performed by the professional software of the Computer-Aided Similarity Evaluation System for Chromatographic Fingerprint of TCM (China Committee of Pharmacopeia, 2004A), which was recommended by the SFDA. The software was employed for synchronization and quantitative comparison between different samples [37].

In addition, the RRT and RPA of each characteristic peak related to the reference peak (*R,S*-goitrin) were also statistically analyzed using the SPSS software package (version 15.0; IBM: Chicago, IL, USA, 2006) and Soft Independent Modeling of Class Analogy (SIMCA)-P (version 13.0; Umetrics: Basel, Switzerland, 2012) [39–41].

## 4. Conclusions

In conclusion, the UPLC fingerprint and quantitative analysis of the water-soluble extract of *Radix Isatidis* were first established in this study to determine the eight bioactive constituents in *Radix Isatidis* and its finished products.

Meanwhile, the results from the three chemometric techniques (SA, HCA, and PCA) showed good consistency with one another. The samples from different locations of the same type (crude herbs or prepared slices) were still clustered into one group, and the eight bioactive components were mostly close, which indicates the similarity of internal quality of the samples in the same type. Furthermore, eight marker constituents were found to be specific variables, which could provide the most discrimination and quality control of *Radix Isatidis* and its finished products by quantitative analysis. The PCA loading plot identified the greatest impact factor of characteristic constituents to the classification. In addition, this chromatographic method was efficient and rapid in distinguishing *Radix Isatidis* from other species such as NBLG.

Based on this study, the UPLC fingerprint and quantitative analysis methods provided reliable assurance on systematic and complete quality control on *Radix Isatidis* and its finished products, which would be helpful in improving reasonable developments in *Radix Isatidis* and its finished products.

## Figures and Tables

**Table 1:** Regression data, limits of detection (LODs), and limits of quantification (LOQs) for the eight bioactive constituents.

Compound	Regression equation (*Y* = a*x* + b) a	*R* b	Linear range (μg mL^−1^)	LOD a (μg mL^−1^)	LOQ b (μg mL^−1^)
Hypoxanthine	*Y* = 914.32*x* − 684.64	0.9999	6.50–130.00	0.015	0.049
Uridine	*Y* = 1945.9*x* + 263.11	0.9999	4.12–82.40	0.012	0.041
Adenosine	*Y* = 6870.2*x* − 3669.6	0.9999	4.26–85.20	0.005	0.017
Guanosine	*Y* = 12618*x* − 1461.7	1.0000	3.90–78.00	0.017	0.045
Progoitrin	*Y* = 2579.2*x* − 886.87	0.9997	2.30–34.50	0.563	1.875
Epiprogoitrin	*Y* = 4336.8*x* − 68.733	0.9998	2.30–34.50	0.708	2.100
*R*,*S*-goitrin	*Y* = 1756.6*x* − 757.89	1.0000	4.50–90.00	0.014	0.045
Gluconapin	*Y* = 1865.8*x* − 240.44	0.9999	2.05–30.75	0.456	1.640

a *Y* and *x* stand for the peak area and the injection quantity (μg) of each standard substance, respectively;

b *R* = correlation coefficient, *n* = 6.

**Table 2:** Precision, reproducibility, stability, and recovery of the eight bioactive constituents.

Compound	Precision RSD (%) (*n* = 6)	Reproducibility RSD (%) (*n* = 6)	Stability RSD (%) (*n* = 6)	Recovery (%) (*n* = 9) Mean ± RSD (%)
Hypoxanthine	0.15	0.46	0.22	101.5 ± 1.63
Uridine	0.18	0.10	0.39	101.2 ± 1.33
Adenosine	0.07	0.61	0.49	101.1 ± 1.11
Guanosine	0.12	1.24	1.30	100.9 ± 1.72
Progoitrin	0.11	0.19	0.24	99.5 ± 1.81
Epiprogoitrin	0.11	1.92	1.44	100.2 ± 1.73
*R*,*S*-goitrin	0.48	0.72	0.91	100.4 ± 0.73
Gluconapin	0.31	0.33	0.48	103.0 ± 1.14

**Table 3:** The contents (mg g^−1^) of eight targets in 21 commercial samples (*n* = 3).

Name	No.^a^	Content b (mg g^−1^)								

		1 c	2	3	4	5	6	7	9	Total
Prepared slices	S-01	0.137	0.414	5.938	6.605	0.492	0.525	0.776	4.051	18.937
	S-02	0.022	0.279	1.529	1.500	0.293	0.377	0.899	0.870	5.768
	S-03	0.042	0.436	2.389	2.094	0.343	0.465	0.789	1.210	7.767
	S-04	0.024	0.283	2.465	2.293	0.342	0.336	0.414	1.190	7.347
	S-05	0.019	0.278	3.331	3.355	0.347	0.345	0.551	1.977	10.203
	S-06	0.037	0.416	1.178	1.413	0.372	0.424	0.732	0.401	4.974
	S-07	0.021	0.384	1.416	1.750	0.336	0.347	0.483	0.574	5.310
	S-08	0.336	0.345	1.950	2.441	0.348	0.379	0.436	0.501	6.735
Crude herbs	S-09	0.056	0.083	5.929	6.605	0.114	0.095	0.057	10.020	22.959
	S-10	0.022	0.057	7.345	6.565	0.127	0.132	0.083	9.458	23.790
	S-11	0.028	0.070	5.286	6.424	0.120	0.116	0.070	10.130	22.244
	S-12	0.035	0.084	3.934	5.660	0.124	0.137	0.123	7.235	17.333
	S-13	0.048	0.057	6.396	6.534	0.105	0.135	0.062	6.747	20.084
	S-14	0.040	0.059	5.507	6.253	0.122	0.327	0.084	9.611	22.003
	S-15	0.033	0.070	6.628	5.713	0.105	0.207	0.054	5.498	18.309
Granules	S-16	N.D. d	0.045	N.D.	N.D.	0.006	0.008	0.085	0.330	0.473
	S-17	0.004	0.022	N.D.	N.D.	N.D.	N.D.	0.027	0.094	0.147
	S-18	N.D.	0.058	N.D.	N.D.	0.003	0.005	0.075	0.576	0.716
	S-19	N.D.	0.021	N.D.	N.D.	0.001	N.D.	0.033	0.100	0.154
	S-20	0.006	0.061	N.D.	N.D.	0.007	0.006	0.087	0.515	0.681
	S-21	0.036	0.053	N.D.	0.102	0.012	0.010	0.067	0.169	0.448

a No. means sample numbers as listed in Table 4;

b Values in mg g^−1^ of dry raw materials; and in mean ± SD, *n* = 3, the SD was < 4% of the mean, which is not shown for clarity;

c (1–7,9) mean the bioactive constituents number as listed in Figure 1;

d N.D.: below LOD.

**Table 4:** All samples used in this work and their corresponding similarities.

Name	No.	Batch No.	Resource	Origins	Similarity
**Prepared Slices**	S-01	20101121	Anhui	*Isatis indigotica* Fort.	0.991
	S-02	101001	Anhui	*Isatis indigotica* Fort.	0.933
	S-03	8100182	Anhui	*Isatis indigotica* Fort.	0.948
	S-04	100709	Anhui	*Isatis indigotica* Fort.	0.947
	S-05	20110213	Jiangsu	*Isatis indigotica* Fort.	0.967
	S-06	100713	Mongolia	*Isatis indigotica* Fort.	0.954
	S-07	100602	Mongolia	*Isatis indigotica* Fort.	0.954
	S-08	101120	Zhejiang	*Isatis indigotica* Fort.	0.967

**Crude herbs**	S-09	20110224-1	Shanghai	*Isatis indigotica* Fort.	0.777
	S-10	20110224-2	Shanghai	*Isatis indigotica* Fort.	0.774
	S-11	20110224-3	Shanghai	*Isatis indigotica* Fort.	0.752
	S-12	20080902	Harbin	*Isatis indigotica* Fort.	0.759
	S-13	blg-081020	Henan	*Isatis indigotica* Fort.	0.818
	S-14	20110205	Anhui	*Isatis indigotica* Fort.	0.820
	S-15	20081017	Hebei	*Isatis indigotica* Fort.	0.811

**Granule**	S-16	090921	Shanghai	-	-
	S-17	100302	Shanghai	-	-
	S-18	A0F090	Guangzhou	-	-
	S-19	L9F043	Guangzhou	-	-
	S-20	100303	Sichuan	-	-
	S-21	100702	Sichuan	-	-

***Nan-Banlangen***	S-22	060308-1	Jiangxi	*Baphicacanthus eusia* (Nees) Bremek.	-
	S-23	1678-3	Guizhou	*Baphicacanthus eusia* (Nees) Bremek.	-
	S-24	20110923	Fujian	*Baphicacanthus eusia* (Nees) Bremek.	-
	S-25	20111025	Fujian	*Baphicacanthus eusia* (Nees) Bremek.	-
